# Priority Colonization of Endophytic Fungal Strains Drives Litter Decomposition and Saprotroph Assembly via Functional Trait Selection in Karst Oak Forests

**DOI:** 10.3390/microorganisms13051066

**Published:** 2025-05-03

**Authors:** Dongmei Yang, Zaihua He, Yonghui Lin, Xingbing He, Xiangshi Kong

**Affiliations:** 1 College of Biology and Environmental Sciences, Jishou University, Jishou 416000, China; 2 Hunan Provincial Key Laboratory of Ecological Conservation and Sustainable Utilization of Wulingshan Resources, Jishou University, Jishou 416000, China; 3 College of Tourism and Management Engineering, Jishou University, Zhangjiajie 427000, China; kongxiangshi@126.com

**Keywords:** endophyte, mass loss, microbial diversity, microbial community assembly, microbial co-occurrence network

## Abstract

Litter decomposition dynamics are largely governed by microbial interactions. While the involvement of endophytic fungi in early-stage decomposition and microbial succession is well established, their species-specific contributions to decomposer community assembly remain insufficiently understood. This study investigated the effects of single-strain endophytic colonization using dominant species (*Tubakia dryina*, *Tubakia dryinoides*, *Guignardia* sp.) and rare species (*Neofusicoccum parvum*, *Penicillium citrinum*) on *Quercus acutissima* leaf decomposition through a controlled field experiment in a karst ecosystem. Endophytes accelerated decomposition rates across treatments but paradoxically reduced transient CO_2_ emissions, linked to intensified microbial carbon and phosphorus limitations in late stages. Contrary to expectations, decomposition efficiency was governed by endophytic fungal species traits rather than colonization abundance, with rare species outperforming dominant taxa. Endophytes induced significant fungal community restructuring, reducing *Ascomycota* while enriching lignin-degrading *Basidiomycota*, but minimally affected bacterial composition. Co-occurrence networks revealed endophyte-driven fragmentation of microbial connectivity, with only two keystone fungal hubs (*Trechispora* sp. and *Russula carmesina*) identified compared to natural communities. Endophytic colonization improved fungal community assembly, mediated by an increase in lignin-degrading *Basidiomycota* and the suppression of pathogenic *Leotiomycetes* lineages. Our findings demonstrate that endophytes hierarchically regulate decomposer communities through phylogenetically conserved fungal interactions, prioritizing functional trait selection over competitive dominance, thereby stabilizing decomposition under nutrient constraints. This mechanistic framework advances predictions of litter decay dynamics in forest ecosystems undergoing microbial community perturbations.

## 1. Introduction

Litter decomposition is a cornerstone of forest ecosystem dynamics, driving nutrient cycling, carbon sequestration, and energy flow through microbial food webs [1]. This intricate process of organic matter breakdown is primarily mediated by microorganisms, with fungi and bacteria being the most active decomposers [2]. These microbial communities are essential for releasing nutrients back into the soil and play a critical role in maintaining soil structure and fertility. Recent research has unveiled the significant involvement of endophytic fungi in litter decomposition, expanding their ecological role beyond living plant tissues [3]. Once considered solely as symbionts within the phyllosphere, endophytes are now recognized as pioneers in the microbial assembly history of plant litter decomposition, influencing the rate and efficiency of carbon release and nutrient recycling [4,5,6].

The role of endophytic fungi in litter decomposition is multifaceted. They produce a range of extracellular enzymes that break down complex organic compounds such as cellulose, hemicellulose, and lignin. This enzymatic activity is crucial for the initial stages of decomposition, where easily accessible nutrients are rapidly consumed [7]. Additionally, endophytic fungi can alter the physical structure of leaf litter, increasing the surface area available for colonization by other microbes and thus accelerating decomposition [8]. The presence of endophytic fungi can also influence the palatability of litter to other decomposers, potentially through the production of secondary metabolites that can inhibit or promote the growth of other microorganisms [9].

The concept of historical assembly in ecology underscores the importance of species arrival order and their interactions in shaping community structure and ecosystem function [10]. In the context of litter decomposition, the sequential colonization by microbes, including pioneer endophytic fungi, can have profound effects on the decomposition process [11]. This phenomenon, known as priority effects, suggests that early-arriving species can alter conditions in a way that influences the establishment and performance of later-arriving species [12,13]. Endophytic fungi, due to their pre-existing relationship with plant tissues, can gain a competitive advantage as pioneer colonizers, thereby potentially modulating the succession of decomposer communities and the overall decomposition trajectory [14,15]. We hypothesize that priority colonization by endophytic fungi will significantly influence the structure and function of the decomposer community, leading to alterations in decomposition rates and nutrient cycling patterns.

In ecosystems, dominant species are those that occupy a leading position in the community due to their high abundance, large biomass, or significant ecological functions [16]. These species typically possess larger niches, enabling them to dominate the community through competition and resource utilization [17]. Dominant species often exhibit strong competitive abilities and play crucial roles in community assembly and stability [17]. By occupying key resources and spatial niches, they influence the survival and reproduction of other species. This competitive edge allows dominant species to maintain their dominant position in the ecosystem and enhance ecosystem resilience through selection effects and by promoting asynchrony among species [18,19]. Dominant species not only dominate in terms of quantity but also play a key role in ecosystem functions, reflecting the community’s adaptability and competitiveness in the environment, as well as representing the stability of the community.

Dominant species play a central role in ecosystem functions by directly influencing ecosystem processes and trajectories through alterations in environmental conditions and resource availability [20]. For example, changes in dominant species in plant communities can significantly alter community productivity and ecosystem responses to environmental gradients [21]. In microbial communities, dominant species often drive biogeochemical cycles and maintain ecosystem stability under disturbances [22,23]. Despite the extremely high diversity of soil microbial communities, only a small proportion of species can be considered dominant. These species are highly abundant across most soils and drive ecosystem responses to extreme climatic conditions [24,25]. These dominant species play a crucial role in shaping ecosystem responses to environmental changes through their high abundance and wide distribution compared to rare species [26,27,28]. Therefore, we propose the second hypothesis that species dominance can regulate the priority effects of endophytic fungi, not only shaping microbial community composition but also influencing litter decomposition functions through interactions with subsequent species.

Karst ecosystems are characterized by shallow soils, high rock exposure, intermittent hydrology, and pronounced nutrient limitations, particularly of nitrogen and phosphorus. These environmental constraints make karst forests highly sensitive to microbial regulation and ideal systems for investigating the role of microbial interactions in decomposition [29]. Oak (*Quercus* spp.) forests dominate large areas of subtropical karst landscapes and generate a significant amount of recalcitrant litter, providing a suitable substrate for studying how endophytes influence decomposition under nutrient stress. Although the role of endophytic fungi in decomposition has been explored in other forest types, such as coniferous and tropical forests, few studies have targeted karst systems. This study therefore provides new insights into microbial assembly and ecosystem function under unique edaphic and environmental stress conditions.

Under the framework of historical assembly theory, this study combined controlled indoor priority colonization with field decomposition experiments to evaluate how the priority effects of endophytic fungal strains influence the structure and function of decomposer communities in saprotrophic systems, thereby shaping litter decomposition dynamics. This research deepens our understanding of species-specific and trait-driven mechanisms by which leaf endophytic fungi contribute to forest litter decomposition, with particular significance under nutrient-limited conditions in karst ecosystems.

## 2. Materials and Methods

### 2.1. Experimental Site and Samples Collection

The experimental site is located in the oak (*Q*. *acutissima*) forest of Zhailong Village (25°52′–31°24′ N and 107°4′–112°2′ E), Jishou City, in the Wuling Mountain region of China. The region is characterized by a subtropical monsoon climate, with a mean annual temperature of 12–17 °C and annual precipitation ranging from 1100 to 1600 mm. The topography predominantly consists of mountainous and hilly terrain, featuring steep slopes and complex geological formations. These unique habitat conditions foster remarkable biodiversity, supporting diverse subtropical forest ecosystems including broad-leaved and coniferous forests. This study focuses on the leaf litter of *Q. acutissima*, a dominant species in this area. As a principal contributor to soil nutrient inputs, its leaf litter plays a critical role in improving regional soil fertility and driving carbon cycling processes in this ecosystem [30].

Leaf samples were collected at the apex of the leaf-fall season in November 2023. To ensure the leaves remained uncontaminated by soil, we employed a method of gently agitating the oak trees to dislodge the freshly fallen leaves, which was then carefully intercepted using suspended nylon nets at the height of 1.5 m. A subset of freshly fallen leaves was collected and immediately placed into sterile, resealable plastic bags to maintain sterility. These bags, covered with ice to preserve their freshness, were swiftly transported to the laboratory. Upon arrival, the samples were stored at −4 °C to inhibit microbial growth and preserve the endophytic community for the isolation of endophytic fungi. Another subset of the freshly fallen leaves was returned to the laboratory and subjected to drying at 50 °C until they reached a constant weight as substrates for subsequent decomposition experiments.

### 2.2. Isolation of Endophytic Fungi

**Figure 1:**
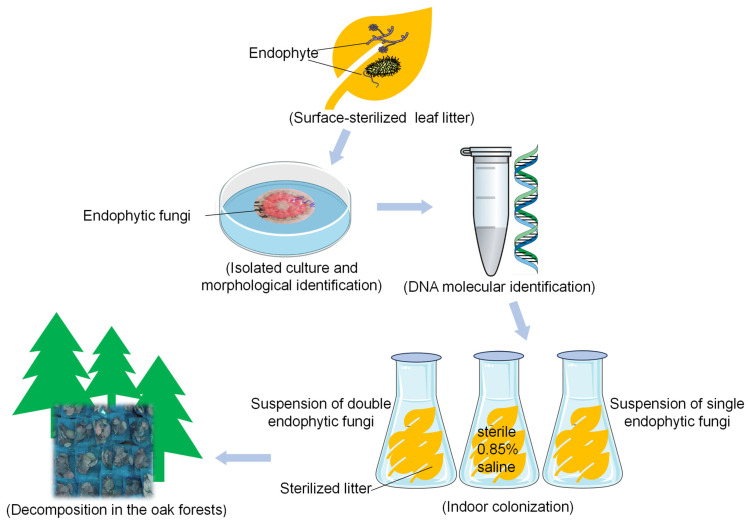
Schematic diagram of the experimental design.

Above collected freshly fallen leaves for isolating endophytic fungi were manipulated according to the procedure of He et al. [5]. The surface-sterilized leaves were cut with sterile knives into small pieces of about 3 mm width. All pieces (one piece per plate) were placed on 2% agar medium containing 20% potato and 1% dextrose (PDA) for endophyte isolation, and then were incubated at 25 °C about 3–7 days with frequently monitoring the growth of colonies at the incisions (Figure 1). Each colony was purified on PDA medium and all isolated fungal colonies were classified into morphotypes by colony-forming characters, e.g., color, shape, growth-rate and pigments. Actively growing mycelium of representative isolates per morphotype were further identified by ITS gene sequencing using the primers ITS1/ITS4 as described by Gardes and Bruns [31]. The detailed reaction procedure is seen in the research of He et al. [5]. The sequencing procedure was achieved at BIOZERN Biotech. Co., Ltd. (Shanghai, China). The sequences were compared with the ITS gene sequences available in the Genebank nucleotide library using BLAST (version 2.14.0) with a close phylogenetic distance with >97% similarity. Ultimately, a total of 2081 fungal isolates were classified into 45 distinct morphotypes, and were identified as 17 species at the operational taxonomic unit (OTU) level (Table 1).

### 2.3. Field Experiment Design for Litter Decomposition

Based on the abundance of isolated endophytic fungi, we selected the top three dominant species, namely *Tubakia dryina* QA01 (abbreviated as Td01), *Tubakia dryinoides* QA02 (abbreviated as Tdr02), and *Guignardia* sp. QA03 (abbreviated as Gs03), as the target dominant endophytic strains. Additionally, we chose the bottom two rare species, *Penicillium citrinum* QA17 (abbreviated as Pc17) and *Neofusicoccum parvum* QA16 (abbreviated as Np16), as references. To investigate whether the priority effects of dominant strains are influenced by rare strains, we established three strain combinations, pairing each of the three dominant strains with one of the selected rare strains. *P. citrinum* is the only isolate detected in our previous amplicon sequencing data and has persisted throughout a one-year litter decomposition experiment, from the initial to the later stages, indicating its sustainability in the decomposition process despite its low abundance [30]; Meanwhile, *P. citrinum* is capable of producing various plant cell wall-degrading enzymes, such as cellulases and xylanases, and possesses the potential to interact with or antagonize other microorganisms through secondary metabolites. Therefore, it may play a dual role in litter decomposition by contributing to cooperative degradation and modulating the microbial community. Based on the above comprehensive considerations, we selected the rare strain Pc17 to combine with the three dominant strains (Td01-Pc17, Tdr02-Pc17, and Gs03-Pc17).

The inoculum preparation process was as follows: each of the aforementioned endophytic fungi was individually inoculated into a potato dextrose liquid medium. The medium was prepared by cooking 200 g of fresh potatoes in water for 30 min, filtering the cooking liquid through four layers of gauze, adding 20 g of dextrose (MACKLIN, Shanghai, China) to the filtrate, and diluting the mixture to a final volume of 1 L with distilled water. The fungi were cultured for 5 days to produce mycelial biomass. The mycelium was then thoroughly washed with sterile distilled water until all residual medium was removed. Subsequently, the mycelium was gently disrupted using a sterile glass rod to create a fungal suspension in sterile saline solution (0.85% NaCl, MACKLIN, Shanghai, China), which was used as the inoculum for pre-colonization when required.

The freshly collected litter was first sterilized with gamma rays at a dosage of >22 kGy. The sterilized litter was then placed into sterile Erlenmeyer flasks, followed by inoculation with either the individual endophytic fungi or combinations of two endophytic fungi prepared as described above. For each flask containing litter, 20 mL of the endophyte inoculum (or a combination of two fungi at a 1:1 volume ratio) was added and incubated for 7 days at 25 °C to allow colonization by the endophytes. Meanwhile, a blank control without any fungal inoculation was also set up by adding 20 mL of sterile 0.85% saline solution.

The litter from all the different inoculation treatments was transferred into litterbags (1 mm nylon mesh, 20 cm × 20 cm dimensions) and placed in the litter layer under the oak forest on 30 April 2024 for decomposition. Each treatment (including the uninoculated control) was replicated with 10 litter bags, and bags of the same treatment were placed within a 2 m × 2 m plot. The distance between plots of different treatments did not exceed 5 m to minimize the impact of soil heterogeneity. After the field decomposition experiment, the samples were collected on 6 November 2024. The samples were kept on ice and promptly transported to the laboratory, where five were used for microbial sequencing and enzyme activity assays, and the other five were used for measuring non-active indicators.

### 2.4. Extracellular Enzyme Activity, Carbon Dioxide Release, and Mass Loss

Leaf litter samples were processed by removing adhered soil, followed by fragmentation and homogenization in a buffer solution using a mortar and pestle. The homogenate was centrifuged (12,000× *g*, 10 min) to collect supernatants for enzyme assays. Activities of β-glucosidase (βG), exocellulase (C1), and endocellulase (Cx) were determined via the 3,5-dinitrosalicylic acid (DNS, MACKLIN, Shanghai, China) method [32]. For β-N-acetylglucosaminidase (NAG) and acid phosphatase (AP), enzymatic hydrolysis of p-nitrophenyl (pNP) derivatives (pNPN-acetyl-4-D-glucosaminide and pNP-phosphate, respectively, MACKLIN, Shanghai, China) was quantified spectrophotometrically at 405 nm [33,34]. Leucine aminopeptidase (LAP) activity was assessed using L-leucine-p-nitroanilide (MACKLIN, Shanghai, China) as the substrate, with absorbance measured at 410 nm [35]. All enzyme activities were normalized as μM hydrolyzed product per gram dry litter per hour (μM g^−1^ h^−1^).

CO_2_ releases were measured by incubating 0.5 g litter in sealed flasks at 25 °C for 48 h in darkness. Released CO_2_ was trapped in 0.5 M NaOH (MACKLIN, Shanghai, China) and quantified via two-phase titration with 0.05 M HCl (MACKLIN, Shanghai, China) [36], expressed as mmol CO_2_ g^−1^ dry litter day^−1^.

Mass loss was calculated by comparing initial and post-exposure dry weights. Surface-cleaned litter samples from five replicate bags per treatment were oven-dried (50 °C to constant weight) at each sampling interval. Data represent the percentage of remaining mass relative to initial dry mass.

### 2.5. DNA Extraction, PCR Amplification, and NovaSeq Sequencing

Total genomic DNA was extracted from litter samples using the E.Z.N.A.^®^ Soil DNA Kit (Omega Bio-Tek, Norcross, GA, U.S.). DNA concentration and integrity were verified using a NanoDrop 2000 spectrophotometer (Thermo Fisher Scientific, USA) and 1% agarose gel electrophoresis (Biowest, Spain), respectively. For fungal community analysis, the ITS1 region was amplified with primers ITS1F (5′-CTTGGTCATTTAGAGGAAGTAA-3′) and ITS2R (5′-GCTGCGTTCTTCATCGATGC-3′), while bacterial communities were characterized by amplifying the 16S V5-V7 region using primers 799F (5′-AACMGGATTAGATACCCKG-3′) and 1193R (5′-ACGTCATCCCCACCTTCC-3′). PCR reactions were performed on a GeneAmp^®^ 9700 System (ABI, USA), followed by library preparation and paired-end sequencing (2 × 250 bp) on an Illumina NovaSeq platform (BIOZERN Biotech, Shanghai, China). The detailed processes of PCR and library construction are found in Zhao et al. [37].

Raw sequencing reads were quality-filtered using Trimmomatic v0.39 to eliminate low-quality bases. Overlapping paired-end reads were merged with FLASH, and operational taxonomic units (OTUs) were clustered at 97% sequence similarity using UPARSE (v10), with chimera removal via UCHIME. Taxonomic classification of fungal ITS and bacterial 16S rRNA sequences was performed against the UNITE (v8.2) and SILVA (SSU138.1) databases, respectively, using the uclust algorithm (80% confidence threshold). Sequencing data are publicly accessible under NCBI BioProject PRJNA1231035.

### 2.6. Leaf Litter Extracellular Enzyme Stoichiometry (EES)

To evaluate microbial resource acquisition strategies for carbon (C), nitrogen (N), and phosphorus (P) resources, we analyzed the stoichiometric ratios of C, N, and P based on the relative activities of key exoenzymes (such as β-glucosidase (βG), acid phosphatase (AP), N-acetyl-β-glucosaminidase (NAG), and leucine aminopeptidase (LAP)) using quantitative vector analysis [38,39]. Vector length (dimensionless) reflects the relative C limitation, with higher values indicating stronger C constraints. Vector angle (°) distinguishes between N (<45°) and P (>45°) limitations [40,41,42]. The calculations for vector length and angle are as follows:

$$\begin{array}{c} \mathrm{Vector}\;\mathrm{length}=\sqrt[{2}]{[\frac{Ln(BG)}{Ln(NAG+LAP)}{]}^{2}+[\frac{Ln(BG)}{Ln(AP)}{]}^{2}}\\ \mathrm{Vector}\;\mathrm{angle}=\mathrm{Degrees}\left\{ATAN2\left[\frac{Ln(BG)}{Ln(AP)}\right],\left[\frac{Ln(BG)}{Ln(NAG+LAP)}\right]\right\} \end{array}$$

In the equation, ATAN2 represents the angle of the arc tangent from the origin to the point (Ln BG/Ln AP, Ln BG/Ln (NAG + LAP)), and “Degrees” indicates the tangent angle.

The ratios of C:N, C:P and N:P acquisition enzymes can reveal differences in relative resource allocation for the acquisition of C, N, and P [38]. The ratios of enzyme C, N, and P (E_C:N_, E_C:P_, and E_N:P_) are calculated using the following formulas:

$$\begin{array}{l} E_{C:N}=\frac{\mathrm{Ln}(\mathrm{BG})\;}{\mathrm{Ln}(\mathrm{NAG}+\mathrm{LAP})\;}\\ E_{C:P}=\frac{\mathrm{Ln}(\mathrm{BG})\;}{\mathrm{Ln}(\mathrm{AP})\;}\\ E_{N:P}=\frac{\mathrm{Ln}(\mathrm{NAG}+\mathrm{LAP})\;}{\mathrm{Ln}(\mathrm{AP})\;} \end{array}$$

### 2.7. Leaf Litter Organic Matter Quality

Fourier-transform infrared (FTIR, Shimadzu, Japan) spectroscopy (Perkin Elmer Spectrum Two) was employed to assess litter decomposition dynamics. Samples mixed with KBr (1:100 w/w) (MACKLIN, Shanghai, China) were scanned at 4 cm^−1^ resolution (32 scans, 4000–400 cm^−1^ range) [43]. Spectral bands corresponding to polysaccharides (C-O), aliphatic (C-H), and aromatic (C=C, C-H) functional groups were analyzed. Two indices were calculated:

Index I (aromatic-to-aliphatic ratio) reflects decomposition extent, with higher values indicating advanced degradation.

Index II (carbon-to-oxygen functional group ratio) quantifies recalcitrance, where increased values correlate with reduced biodegradability [41,44].

The calculations for Index I and Index II are as follows:

$$\begin{array}{l} \mathrm{IndexI}=\frac{{\mathrm{RAISB}}_{\mathrm{aromatic}\;\text{C=C+aromatic}\;\text{C-H}}}{{\mathrm{RAISB}}_{\mathrm{aliphatic}\;\text{C-H}}}\\ \mathrm{IndexII}=\frac{{\mathrm{RAISB}}_{\mathrm{aliphatic}\;\text{C-H+aromatic}\;\text{C=C+aromatic}\;\text{C-H}}}{{\mathrm{RAISB}}_{\mathrm{polysaccharide}\;\text{C-O}}} \end{array}$$

### 2.8. Statistical Analysis

The ANOVA analysis followed by Duncan’s multiple comparisons was employed to compare the significant differences among treatments in enzyme activity, carbon dioxide release, mass loss, organic matter quality, enzyme vector angles, enzyme vector lengths, and EES. Kruskal–Wallis test was used to analyze the differences in microbial alpha diversity, microbial abundance in module of co-occurrence network, and βNTI value of community assembly among treatments. Subsequent post hoc analysis was conducted using Dunn’s test to identify specific differences between treatment groups. Additionally, Spearman correlation analysis was used to explore the relationship between observed variables. A significance level of *p* < 0.05 was considered statistically significant for all analyses. All analyses were conducted using R version 4.3.3.

#### 2.8.1. Microbial Diversity

Alpha diversity indices (Shannon and ACE) for bacterial and fungal communities were computed from operational taxonomic unit (OTU) richness and phylogenetic data using the vegan package in R v4.3.3. Beta diversity was evaluated via principal coordinate analysis (PCoA) based on Bray–Curtis dissimilarity matrices generated from standardized OTU abundances. Community composition differences were statistically tested using permutational multivariate analysis of variance (PERMANOVA) with 999 permutations, implemented through the adonis2 function in vegan [45].

#### 2.8.2. Microbial Community Assembly

To quantify the priority effects of endophytic colonization on decomposer community assembly, a null model framework was applied [46]. Phylogenetic turnover was assessed using the beta Nearest Taxon Index (βNTI), while the Raup–Crick index (RCI) was computed to evaluate compositional variation independent of richness differences. Both metrics were derived from operational taxonomic unit (OTU) abundance and phylogenetic data via the NST package in R. Deterministic and stochastic processes were partitioned based on βNTI and RCI thresholds. Deterministic dominance: |βNTI| > 2, categorized as heterogeneous selection (βNTI > +2) or homogeneous selection (βNTI < −2). Dispersal processes: |βNTI| < 2 and |RCI| > 0.95, distinguishing homogenizing dispersal (RCI < −0.95) from dispersal limitation (RCI > +0.95). Stochastic drift (referred to as “undominated” process): |βNTI| < 2 and |RCI| < 0.95 [46,47]. Detailed description was seen in the research of Yang et al. [30].

#### 2.8.3. Network Analysis

To delineate keystone taxa and microbial interactions during decomposition, co-occurrence networks were constructed for each treatment. Fungal and bacterial OTUs with relative abundances <0.01% or occurrence in <80% of samples were excluded to minimize spurious correlations. Spearman rank correlations between OTUs were computed using the Hmisc package in R (version 4.3.3), with false discovery rate (FDR) correction applied (Benjamini-Hochberg method). Robust correlations (*r* ≥ 0.8, *p* < 0.01) were retained [48]. Undirected networks were generated via the igraph package, and topology parameters (e.g., degree, betweenness centrality) were extracted. Networks were visualized in Gephi (version 0.10.1) using the Fruchterman-Reingold algorithm [49].

Network modularity was assessed using the greedy optimization algorithm (igraph package), identifying modules as densely interconnected subnetworks with sparse inter-module links [50]. Keystone OTUs were defined as those within the top 1% of node degree centrality, reflecting their pivotal roles in maintaining network structure [51,52].

#### 2.8.4. Differential Taxa Identification via LEfSe

Microbial biomarkers distinguishing treatment groups were identified using Linear Discriminant Analysis Effect Size (LEfSe) [53]. The analysis first detected taxa with significant abundance differences (Kruskal–Wallis test, *p* < 0.05), followed by pairwise validation via Wilcoxon rank-sum tests. Linear discriminant analysis (LDA) was then applied to estimate effect sizes, quantifying each taxon’s contribution to group differentiation [54]. Thresholds of LDA score ≥ 4.7 (fungi) and ≥ 3.0 (bacteria) were applied to filter biomarkers [55]. All analyses were implemented in R (version 4.3.3), using the microeco package.

#### 2.8.5. Correlation Analyses of Endophytic Fungal Colonization to Microbial Functions

We employed piecewise structural equation modeling (piecewiseSEM) to evaluate the direct and indirect relationships among endophyte priority colonization, microbial diversity, microbial network, microbial community assembly, degrading enzyme, mass loss, and soil moisture. We initially constructed a prior model that included all hypothesized pathways and iteratively simplified the model by removing non-significant pathways until the final model was achieved. The suitability of the final model was evaluated utilizing Fisher’s C statistic, as implemented in the piecewiseSEM package for R version 4.3.3 [56].

## 3. Results

### 3.1. CO_2_ Release, Mass Loss and Organic Matter Quality

**Figure 2:**
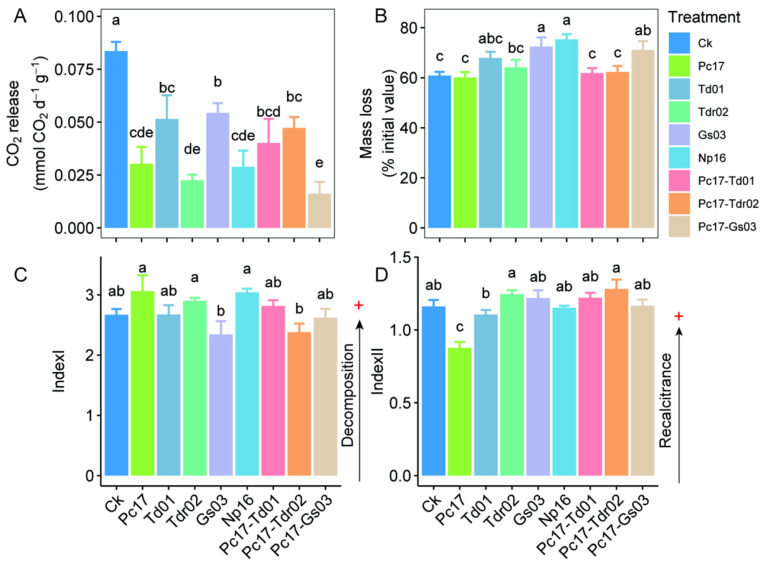
Difference in CO_2_ release (**A**), mass loss (**B**), and organic matter quality (IndexI (**C**) and IndexII (**D**)) at the end of litter decomposition under different endophytic fungal colonization treatments. Different lowercase letters denote statistically significant differences (*p* < 0.05, Duncan’s test) among treatments. Ck (Control: Gamma-irradiated sterile leaf litter). + indicates the direction of increase or enhancement.

Figure 2A illustrates CO_2_ release at the end of litter decomposition. Compared to the control group, endophytic colonization significantly reduced CO_2_ release (*p* < 0.05). Colonization by the dominant species Td01 and Gs03 resulted in higher CO_2_ release compared to treatments with the rare species Pc17 and Np16 (*p* < 0.05). Mixed colonization of the two dominant species (Td01 and Tdr02) with the rare species Pc17 yielded intermediate CO_2_ release levels between those of the individual Td01, Gs03, and Pc17 treatments. Notably, the mixed colonization of Gs03 and Pc17 exhibited the lowest CO_2_ release.

After half year of field litter incubation, endophytic colonization generally increased mass loss in most cases, although some differences were not statistically significant. Notably, the dominant species Gs03, the rare species Np16, and the Pc17-Gs03 mixed treatment showed the most significant increases in mass loss (*p* < 0.05) (Figure 2B).

Colonization by the two rare species (Pc17 and Np16) and the one dominant species (Tdr02) displayed higher Index I values than other treatments, though overall differences among all treatments were not statistically significant (Figure 2C). Similarly, Figure 2D demonstrates minimal differences in Index II across most treatments, with the exception of Pc17 colonization, which showed significantly lower values.

### 3.2. Extracellular Enzyme Activity and Stoichiometry

Differences in extracellular enzyme activities among endophytic colonization treatments at the end of litter decomposition are shown in Figure S1. Exocellulase activities were significantly higher in the Pc17 and Pc17-Td01 treatments compared to the control (Ck) (*p* < 0.05), while other treatments exhibited significantly lower activities, with the Pc17-Gs03 treatment showing the lowest exocellulase activity (*p* < 0.05; Figure S1A). For endocellulase, Pc17, Pc17-Td01, Pc17-Gs03, and Np16 treatments displayed significantly higher activities than the control (*p* < 0.05), whereas the dominant species treatments (Td01, Tdr02, Gs03) and Pc17-Tdr02 showed significantly lower activities (*p* < 0.05; Figure S1B). A similar pattern to endocellulase was observed for β-glucosidase activity (Figure S1C). For NAG (N-acetylglucosaminidase), all endophyte colonization treatments exhibited significantly lower activities compared to the control, with the lowest values observed in Td01, Gs03, and Np16 treatments (*p* < 0.05; Figure S1D). The Pc17-Td01 and Pc17-Gs03 treatments showed significantly higher LAP (Leucine aminopeptidase) activities than the control (*p* < 0.05), while other treatments did not differ significantly (Figure S1E). Similar to NAG, AP (Alkaline phosphatase) activities were lower in all endophyte treatments compared to the control, with the Gs03 treatment exhibiting the lowest activity (*p* < 0.05; Figure S1E).

**Figure 3:**
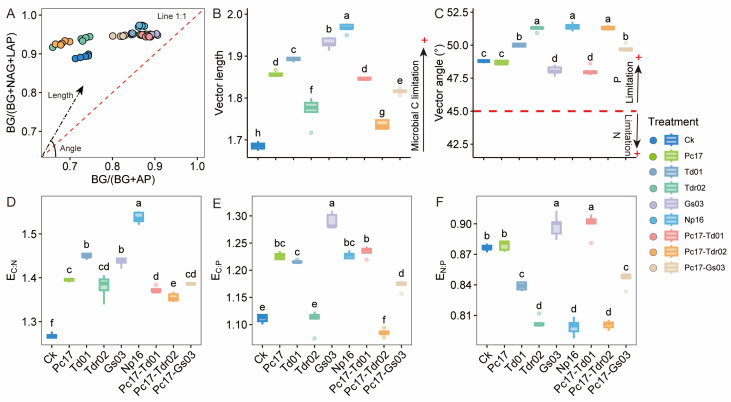
General patterns of microbial carbon, nitrogen, and phosphorus metabolic limitations and stoichiometric characteristics of extracellular enzymes at the end of litter decomposition under different endophytic fungal colonization treatments. (**A**) Enzyme vector model of extracellular enzyme stoichiometry; Vector length (**B**) and angle (**C**) were quantified using the enzymatic vector model based on extracellular enzyme stoichiometry; (**D**) E_C:N_ represents Ln(βG): Ln(NAG+LAP); (**E**) E_C:P_ represents Ln(βG): Ln (AP); (**F**) E_N:P_ represents Ln (NAG+LAP): Ln (AP); βG, β-1,4-glucosidase; NAG, β-1,4-N-acetylglucosaminidase; LAP, leucine aminopeptidase; AP, acid phosphatase. Different lowercase letters denote statistically significant differences (*p* < 0.05, Duncan’s test) among treatments. Ck (Control: Gamma-irradiated sterile leaf litter). + indicates the direction of increase or enhancement.

Enzyme stoichiometry vectors at the end of decomposition varied significantly across treatments (Figure 3A). Vector length and extracellular enzyme C/N and C/P ratios were significantly higher in most endophyte treatments compared to the control (*p* < 0.05), indicating enhanced microbial carbon limitation (Figure 3B,D,E). Carbon limitation was most pronounced in the rare species Np16 treatment, followed by the dominant species Gs03 and Td01, while the Pc17-Tdr02 treatment showed the weakest effect. Vector angle and extracellular enzyme N/P ratios revealed phosphorus (P) limitation across all treatments (Figure 3C,F). Specifically, Td01, Tdr02, Np16, Pc17-Tdr02, and Pc17-Gs03 treatments exhibited stronger P limitation than the control, whereas Gs03 and Pc17-Td01 alleviated P limitation (*p* < 0.05).

### 3.3. Microbial Community Structure and Diversity

In fungal communities, Ascomycota and Basidiomycota were the dominant phyla (Figure S2A). Compared to the control, endophytic colonization significantly reduced the relative abundance of Ascomycota while increasing that of Basidiomycota (Figure S2A). The magnitude of Basidiomycota increase was lowest in the Pc17-Tdr02 treatment, with other colonization treatments showing similar increases. In bacterial communities, Pseudomonadota, Actinomycetota, and Acidobacteriota were the dominant phyla (Figure S2B). Endophytic colonization did not induce significant shifts in bacterial community composition at the phylum level.

**Figure 4:**
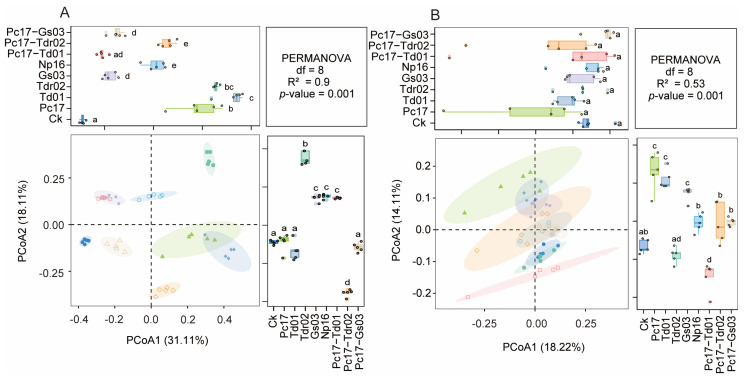
Principal coordinates analysis (PCoA) of fungal (**A**) and bacterial (**B**) communities at the end of litter decomposition under different endophytic fungal colonization treatments. Different lowercase letters denote statistically significant differences (*p* < 0.05, Adonis PERMANOVA) among treatments. Ck (control: gamma-irradiated sterile leaf litter). The ellipses represent the 95% confidence level; the dots represent the samples; The color of the ellipses corresponds to the sample treatment groups.

Principal Coordinate Analysis (PCoA) revealed significant differences in both fungal (Figure 4A) and bacterial (Figure 4B) community structures among all endophytic colonization treatments at the end of incubation (*p* < 0.01), indicating distinct differentiation of microbial communities driven by specific endophyte colonization.

**Figure 5:**
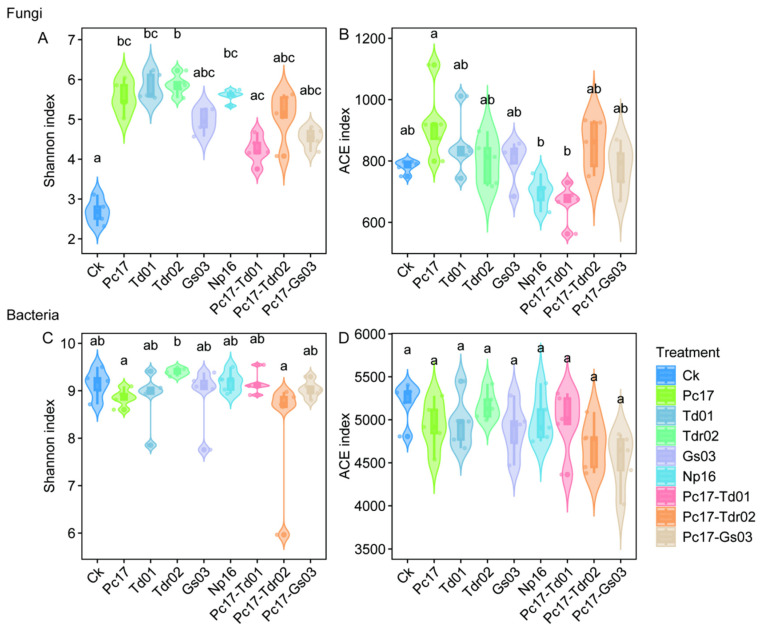
Alpha diversity of fungal (**A**,**B**) and bacterial (**C**,**D**) communities at the end of litter decomposition under different endophytic fungal colonization treatments. Different lowercase letters denote statistically significant differences (*p* < 0.05, Dunn’s test) among treatments. Ck (control: gamma-irradiated sterile leaf litter).

Fungal alpha diversity analysis showed that all endophyte colonization treatments increased the Shannon index, although some differences were not statistically significant (Figure 5A). For the ACE index, significant differences were observed only between Pc17 and Np16, as well as between Pc17 and Pc17-Td01 (*p* < 0.05), with no significant changes in other treatments (Figure 5B). Bacterial alpha diversity remained largely unaffected across treatments, with no significant differences observed in Shannon or ACE indices (Figure 5C,D).

**Figure 6:**
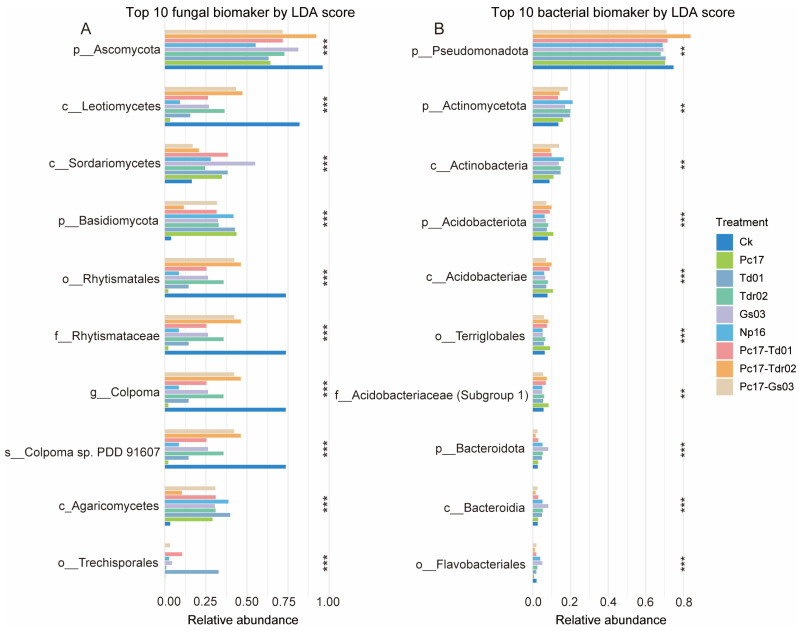
The difference in relative abundance of the top 10 biomarkers for fungi (**A**) and bacteria (**B**) based on LDA scores at the end of litter decomposition under different endophytic fungal colonization treatments. Asterisks denote statistically significant differences (*p* < 0.05, Wilcoxon rank-sum test) among treatments. Ck (control: gamma-irradiated sterile leaf litter).

As shown in Figure S3, 44 fungal (Figure S3A) and 38 bacterial (Figure S3B) clades exhibited statistically significant differences among treatments with an LDA threshold of 4.7 for fungi and 3.0 for bacteria. For both fungi and bacteria, all treatments except Pc17 displayed abundance dominance across various taxonomic levels. Further, we selected the top 10 biomarkers based on LDA scores to analyze differences. For fungal biomarkers (Figure 6A), endophytes significantly reduced the relative abundances of Ascomycote (phylum), Leotiomycetes (class), Rhytismatales (order), *Colpoma* (genus), and *Colpoma* sp. PDD 91607 (species) compared to the control (*p* < 0.01). Conversely, colonization treatments increased the relative abundances of Sordariomycetes (class), Basidiomycota (phylum), and Agaricomycetes (class) (*p* < 0.01). For bacterial biomarkers (Figure 6B), endophytic colonization significantly enhanced the relative abundances of Actinomycetota (phylum), Actinobacteria (class), Bacteroidota (phylum), Bacteroidia (class), and Flavobacteriales (order) compared to the control in most cases (*p* < 0.01).

### 3.4. Microbial Co-Occurrence Network Analysis

**Figure 7:**
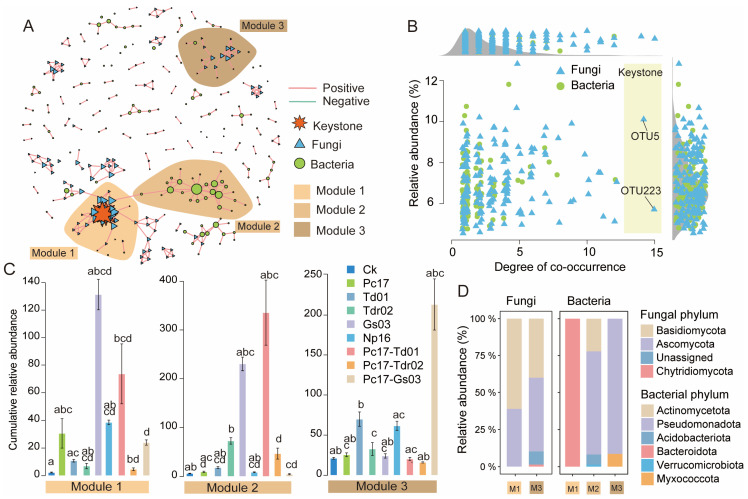
Microbial co-occurrence network analysis at the end of litter decomposition. (**A**) the fungal-bacterial network diagram; (**B**) the co-occurrence and relative abundance of key OTUs; (**C**) the cumulative relative abundance (per million counts (CPM): y-axis × 1000) of microorganisms in the top 3 network modules; (**D**) the relative abundance at the phylum level of fungi and bacteria within the top 3 modules. Different lowercase letters indicate significant differences (*p* < 0.05, Dunn’s test) among treatments. Ck (control: gamma-irradiated sterile leaf litter).

Microbial co-occurrence networks are depicted in Figure 7A, with network analysis identifying three dominant modules: Module 1, Module 2, and Module 3. Based on the degree of co-occurrence, two keystone species, both belonging to fungi, were identified: OTU_5 (*Trechispora* sp.) and OTU_223 (*Russula carmesina*) (Figure 7B). The cumulative relative abundances of species within each module were analyzed across treatments, as shown in Figure 7C. The results revealed the following patterns: Module 1: Endophytic colonization generally increased species abundance within Module 1, with the Gs03 treatment showing the highest increase, followed by Pc17-Td01. Module 2: Endophytic colonization also increased species abundance in Module 2 in most cases, with the Pc17-Td01 treatment exhibiting the highest increase, followed by Gs03. Module 3: The Pc17-Gs03 treatment caused the largest increase in species abundance within Module 3, followed by Td01 and Np16. In the fungal network, Module 1 and Module 3 were primarily composed of Basidiomycota and Ascomycota at the phylum level, with no fungal distribution observed in Module 2. Within Module 1, the abundance of Basidiomycota was higher than that in Module 3, while Ascomycota showed the opposite trend. In the bacterial network, Module 1 was almost entirely dominated by Bacteroidota, whereas Pseudomonadota was the most abundant phylum in Module 2 and Module 3 (Figure 7D).

### 3.5. Community Assembly

**Figure 8:**
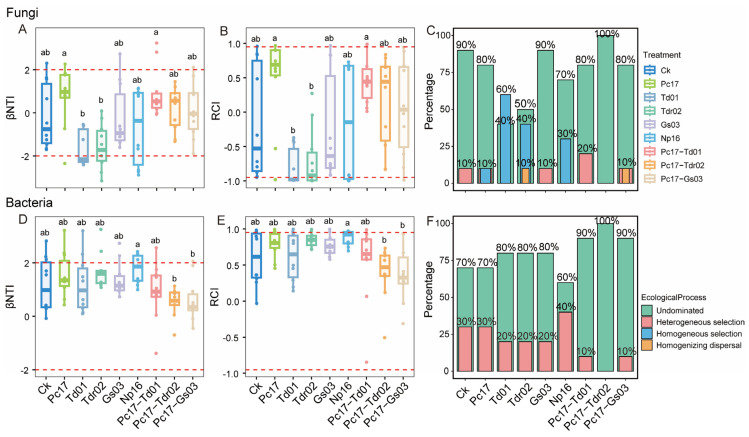
Microbial community assembly process at the end of litter decomposition under different endophytic fungal colonization treatments. (**A**–**C**) Fungi; (**D**–**F**) bacteria. Different lowercase letters indicate significant differences (*p* < 0.05, Dunn’s test) among treatments. Ck (control: gamma-irradiated sterile leaf litter).

For both fungi and bacteria, the absolute values of nearly all βNTI values across different treatments were less than 2, indicating that the community assembly of both fungi (Figure 8A–C) and bacteria (Figure 8D–F) was predominantly driven by stochastic processes (i.e., the sum of undominated and homogenizing dispersal). Among these, homogenizing dispersal, as determined by RCI values, accounted for a very small proportion and was only observed in the fungal community assembly of the Tdr02 and Pc17-Gs03 treatments (Figure 8C). For fungal community assembly, endophytic colonization significantly increased the contribution of deterministic processes (heterogeneous and homogeneous selection) in most cases. The control treatment exhibited a deterministic process contribution of 10%, while the contributions for specific treatments were as follows: Pc17 (20%), Td01 (60%), Tdr02 (50%), Np16 (30%), Pc17-Td01 (20%), and Pc17-Gs03 (20%). Notably, the Gs03 treatment did not increase the deterministic contribution, remaining at 10%, similar to the control. In contrast, the Pc17-Tdr02 treatment showed an increased contribution of stochastic processes, with no detectable deterministic processes (Figure 8C). For bacterial community assembly, endophytic colonization primarily enhanced the contribution of stochastic processes. Compared to the control (70% stochastic processes), only the Np16 treatment significantly increased the deterministic contribution (40%) (Figure 8F).

### 3.6. Relationship of Endophytic Colonization to Microbial Function

**Figure 9:**
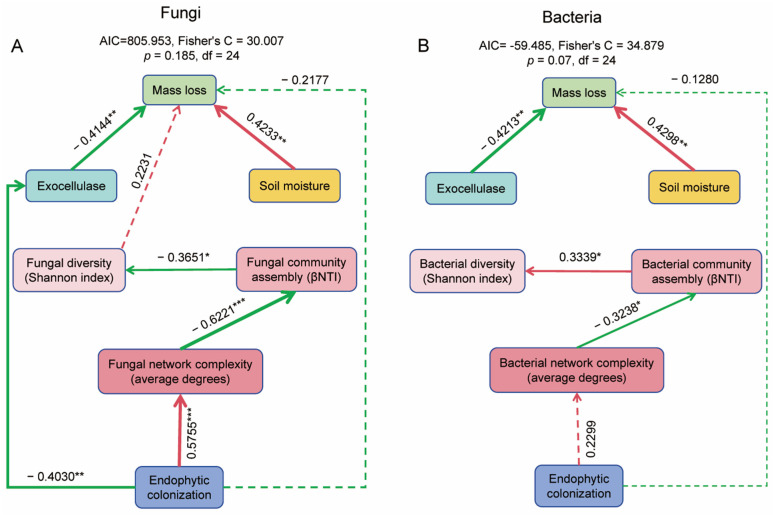
The piecewise structural equation models ((**A**) for fungi and (**B**) for Bacteria) of analyzing the causal relationship between the variables at the end of litter decomposition. Single-headed arrows denote causal relationships. The color of the lines signifies positive effects (red) and negative effects (green), and the solidness of the lines indicates significant (solid line) and non-significant (dashed line) relationships. The values on the lines represent standardized path coefficients. Significance levels for each predictor are denoted as * *p* < 0.05, ** *p* < 0.01, *** *p* < 0.001. Ck (Control: Gamma-irradiated sterile leaf litter).

Structural equation modeling (SEM) analysis (Figure 9) and Spearman’s rank correlation analysis (Figure S4) revealed that endophytic fungal colonization is significantly negatively associated with exocellulase activity, which in turn shows a significantly negative correlation with mass loss rate (*p* < 0.05). Additionally, soil moisture exhibits a significantly positive association with mass loss rate. In terms of community assembly, endophytic fungal colonization demonstrates a significant correlation with fungal network complexity, which is significantly linked to fungal community assembly. Furthermore, fungal community assembly shows a significant association with fungal diversity, indicating a cascading regulatory effect of endophytic fungal colonization (Figure 9A). A similar regulatory pattern is observed for bacterial communities, although some pathways are statistically non-significant in the SEM analysis (Figure 9B). This suggests that endophytic fungal colonization may also modulate bacterial communities through indirect or less pronounced mechanisms.

## 4. Discussion

### 4.1. Effect of Endophytic Colonization on Litter Decomposition

Our findings demonstrate that the priority colonization of endophytic fungi generally enhances litter decomposition across dominant species, rare species, and their mixed colonization treatments, which aligns with our initial hypothesis. The decomposition degree (Index I) and recalcitrance (Index II) of litter also exhibited congruent trends, although the differences among treatments were statistically non-significant. Notably, CO_2_ emission exhibited an inverse response to fungal colonization. This paradoxical pattern may be attributed to accelerated substrate depletion caused by endophyte-mediated decomposition, which likely reduced microbial metabolic activity during later decomposition stages, consequently diminishing transient CO_2_ release. This hypothesis is corroborated by enzyme stoichiometric vector length analysis, revealing that endophyte colonization intensified microbial carbon limitation. Furthermore, phosphorus limitation was exacerbated in most endophytic colonization treatments, with phosphorus—a critical limiting element in litter decomposition—being preferentially depleted [57,58,59]. For instance, Np16 colonization induced the fastest decomposition rate but also caused the strongest phosphorus limitation at the late decomposition stage. The stage-specific phosphorus depletion pattern suggests that early-stage decomposition may benefit from endophytic associations enhancing phosphorus solubilization, whereas late-stage management requires strategic phosphorus supplementation through controlled leaf litter retention or targeted biofertilization. However, not all endophytic colonizations rapidly depleted phosphorus in litter, as exemplified by Gs03 and Pc17-Td01 colonizations that partially alleviated phosphorus limitation. This differential effect highlights the potential utility of employing specific endophyte combinations for ecosystem management or restoration in karst or nutrient-poor ecosystems.

Contrary to our initial expectations, dominant endophytes did not demonstrate superior decomposition rates compared to rare species. Structural equation modeling also indicated a negative correlation between the colonization abundance of endophytic fungi and mass loss rate, though this relationship was not statistically significant. Specifically, colonization by the rare species Np16 achieved the highest decomposition rate, while the dominant species Gs03 exhibited comparable efficiency. This suggests that functional traits of endophytes (e.g., enzymatic capabilities or resource utilization strategies) rather than their abundance predominantly govern decomposition processes [60,61,62]. Colonization by dominant endogenous microbes may maintain basic cellulose decomposition functions through functional redundancy [63,64], while colonization by the rare species Np16, although it did not significantly enhance cellulase activity, resulted in a significantly higher abundance of Basidiomycota in subsequent colonizing microbial communities compared to other treatment groups. This observation suggests that Np16 may promote the decomposition of recalcitrant substances by inducing functional specialization within the microbial community (rather than directly contributing to basic functions). Intermediate mass loss rates in co-colonization treatments of dominant species (Td01, Tdr02, Gs03) and rare species Pc17 imply that inter-species interactions modulate decomposition efficiency through microbial community restructuring. Such regulatory capacity appears partially dependent on fungal abundance hierarchy, reflecting potential ecological trade-offs between competitive dominance and functional complementarity [65].

### 4.2. Effect of Endophytic Colonization on Microbial Composition and Diversity

At the phylum level, the observed decline in relative abundance of *Ascomycota* and concurrent increase in Basidiomycota under endophyte treatments suggests that endophytes may facilitate colonization of functionally specific fungal taxa through niche modulation. Notably, the enrichment of *Basidiomycota*, a phylum renowned for lignin degradation [66], likely contributed directly to the enhanced decomposition of recalcitrant plant residues mediated by endophyte colonization. In contrast, the limited compositional shifts observed in bacterial phyla imply stronger endophyte-driven regulation (e.g., competitive exclusion or metabolic interactions) on fungal communities compared to bacterial communities. This may be because competition among fungi is more inclined to ecological niche partitioning of different types of organic matter, while competition between fungi and bacteria is relatively smaller due to their different ways of resource utilization [67,68,69]. At the diversity level, endophyte colonization significantly altered fungal α-diversity (species richness and evenness), whereas bacterial communities exhibited greater stability, further supporting the hypothesis that fungal-fungal interactions dominate over cross-kingdom (fungal-bacterial) interplay. Notably, β-diversity analysis revealed distinct community differentiation in fungal decomposers under endophyte treatments, while bacterial communities showed limited compositional divergence. This is consistent with the above hypothesis.

We further employed LEfSe analysis to investigate taxonomic-level differences in microbial communities induced by distinct endophyte colonization treatments, applying an LDA threshold of 4.7 for fungi and 3.0 for bacteria. The results revealed significant divergence across multiple taxonomic levels, indicating that endophyte colonization triggered pronounced differentiation of microbial communities during *Quercus acutissima* leaf litter decomposition, implying a strict filtering effect on subsequent colonizing decomposer communities [30]. Among the top 10 biomarkers identified by LDA scores, fungal taxa exhibited substantially greater magnitude of variation in abundance across taxonomic ranks (even under the stringent fungal LDA threshold of 4.7) compared to bacterial taxa, further corroborating the stronger endophyte-mediated impacts on fungal communities. The class *Leotiomycetes*, which encompasses numerous plant pathogens (e.g., powdery mildew-causing *Erysiphales*), displayed suppressed abundance under endophyte colonization despite its dual saprotrophic and pathogenic capabilities. This antagonism likely stems from evolutionary conflicts between endophytes and pathogens, as evidenced by the strongest suppression under *Penicillium* sp. Pc17 treatment—a phenomenon potentially linked to Pc17-derived terpenoid antibiotics disrupting pathogen extracellular enzyme systems [70]. Remarkably, this suppression cascaded through subordinate taxonomic units within *Leotiomycetes*, including the order *Rhytismatales*, family *Rhytismataceae*, and genus *Colpoma* (e.g., *Colpoma* sp. PDD 91607), indicating evolutionarily conserved targeting of specific pathogenic lineages by endophytes. The class Agaricomycetes (representing 53% of Basidiomycota species) mirrored phylum-level responses, underscoring its pivotal role in endophyte-driven enhancement of carbon cycling through lignin decomposition. Collectively, these multi-level responses demonstrate that endophytic fungi implement a “top-down” hierarchical regulation, preferentially restructuring fungal lineages with specific functional attributes. This tiered selection mechanism likely arises from evolutionarily conserved antagonistic/synergistic networks among fungi, enabling stable transmission of interaction patterns from higher (e.g., phylum/class) to lower (e.g., genus/species) taxonomic ranks. In contrast, the absence of analogous cross-kingdom mechanisms between endophytic fungi and bacteria may explain the relative stability of bacterial communities across taxonomic levels [71,72].

### 4.3. Effect of Endophytic Colonization on Microbial Co-Occurrence Network and Community Assembly

The microbial co-occurrence network analysis demonstrated that endophytic colonization restructures the topological organization of decomposer communities, with keystone fungal hubs identified as OTU_5 (*Trechispora* sp.) and OTU_223 (*Russula carmesina*). Notably, these two keystone taxa were absent from the keystone species list from microbial communities during *Quercus acutissima* leaf litter decomposition colonized by naturally endophytic community reported by Yang et al. [30], indicating that microbial succession driven by single-strain endophytic colonization in this study substantially diverges from natural community dynamics. Compared to natural colonization processes [30], specific endophytic colonization generated only two keystone taxa, a marked reduction from natural colonization levels. This suggests that special endophytic colonization induces significant community differentiation, fragmenting previously interconnected species into discrete states. Both keystone species belong to Basidiomycota, implying these keystone species not only contribute directly to litter decomposition but also likely serve crucial functions in maintaining network stability and mediating resource allocation within microbial consortia. Members of *Russula*, recognized as key components in global ectomycorrhizal ecosystems, demonstrate specific associations with *Quercus* species [73]. The elevated abundance of Basidiomycota in Module 1 implies that endophytes may augment functional redundancy among lignin-degrading fungi, potentially stabilizing decomposition processes under environmental fluctuations. Distinct differential patterns observed across modules indicate variable functional impacts from different endophytic species or combinations, highlighting how intrinsic traits of endophytes and their interspecific interactions critically influence microbial community differentiation.

The community assembly analysis revealed that stochastic processes predominantly govern both fungal and bacterial community assembly, consistent with the study of Yang et al. [30]. This suggests that the microbial community assembly in *Quercus acutissima* litter decomposition may primarily be driven by chemical characteristics of the substrate, such as high cellulose content and other readily decomposable components of oak leaves. Stegen et al. [46] similarly reported high variability in microbial succession during litter decomposition. However, endophyte colonization significantly amplified the contribution of deterministic processes in fungal communities (e.g., Td01 and Tdr02 treatments), implying that endophytes exert strong selection pressures on fungal assemblages. This aligns with the hypothesis that endophytes function as ecological filters, shaping microbial community composition through priority effects and niche modification [10,11]. As dominant species, Td01 and Tdr02 likely maintain substantial selective pressures on subsequent microbial colonization, further reinforcing deterministic assembly. Notably, *Tubakia dryinoides* and *T. dryina* exhibit dual ecological roles as both endophytes and reported pathogens of oak trees [74]. Their interactions with saprophytic decomposers in litter decomposition are shaped by evolutionary history. Specifically, antagonism likely arises between *Tubakia* and saprophytic taxa that originated from the same endophytic niche, potentially due to niche overlap or competition for resources during their prior coexistence in plant tissues. This antagonistic legacy drives *Tubakia* to exert strong selection pressures on subsequent microbial colonizers, effectively filtering the assembly of decomposer communities through priority effects and competitive exclusion. Collectively, endophytic fungal colonization markedly influences microbial community assembly, particularly in fungal communities, primarily through restructuring microbial co-occurrence networks, i.e., microbial interactions. This process ultimately cascades to regulate microbial diversity via hierarchical ecological interactions.

## 5. Conclusions

In nutrient-poor karst ecosystems, stress-adapted rare endophytes (e.g., *Neofusicoccum parvum* Np16) drive litter decomposition through microbial specialization and hierarchical restructuring of fungal communities. These endophytes enhance decomposition efficiency by enriching Basidiomycota while suppressing pathogenic lineages (e.g., Leotiomycetes), thereby stabilizing vegetation and nutrient cycling. Critically, rare endophytes outperform dominant taxa in regulating decomposer dynamics, particularly under phosphorus limitation, by prioritizing niche differentiation over resource competition—a key adaptation to oligotrophic soils. Endophytes, especially dominant ones, act as ecological filters, amplifying deterministic assembly in fungal communities through competitive exclusion and functional complementarity. This regulation cascades to microbial co-occurrence networks, where keystone Basidiomycota taxa (e.g., *Russula carmesina*) mediate resource allocation and network stability. In contrast, bacterial communities remain stable due to limited cross-kingdom interactions, without destabilizing bacterial-driven processes like nitrogen fixation. For ecological restoration, we propose the following: (1) prioritizing rare endophytes (e.g., Np16) for early-stage decomposition and phosphorus-mobilizing strains (e.g., Gs03) for late-stage nutrient retention; (2) engineering synthetic consortia to align nutrient release with plant demands; (3) leveraging endophyte-pathogen antagonism (e.g., *Tubakia* suppression) for biocontrol. Future research should elucidate the evolutionary trade-offs governing endophytic functional traits and cross-kingdom interactions, thereby refining restoration strategies to enhance nutrient-use efficiency and stress resilience in vulnerable karst ecosystems.

## Figures and Tables

**Table 1:** Relative abundance of 17 endophytic fungi in *Quercus acutissima* leaves.

Endophytic Fungal Species	Strain Number	Relative Abundance
*Tubakia dryina*	Td01	26.81%
*Tubakia dryinoides*	Tdr02	24.94%
*Guignardia* sp.	Gs03	19.89%
*Aspergillus sydowii*	As04	4.42%
*Aspergillus* sp.	As05	3.65%
*Botryosphaeria dothidea*	Bd06	3.56%
*Phomopsis* sp.	Ps07	3.17%
*Colletotrichum boninense*	Cb08	2.69%
*Acrocalymma medicaginis*	Am09	2.21%
*Bjerkandera adusta*	Ba10	2.16%
*Schizophyllum* sp.	Ss11	1.97%
*Cytospora diatrypelloidea*	Cd12	1.68%
Fungal sp.	Fs13	1.01%
*Colletotrichum gigasporum*	Cg14	0.77%
*Nigrograna hydei*	Nh15	0.48%
*Neofusicoccum parvum*	Np16	0.38%
*Penicillium citrinum*	Pc17	0.19%

## Data Availability

The datasets in this study are available in Supplementary Materials Figures S1–S4.

## Supplementary Materials

The following supporting information can be downloaded at: https://www.mdpi.com/article/10.3390/microorganisms13051066/s1, Figure S1: Changes in the activity of single extracellular enzymes at the end of litter decomposition under different endophytic fungal colonization treatments; Figure S2: Species composition of the top 10 taxa at the phylum level of bacterial and fungal communities under different endophytic fungal colonization treatments; Figure S3: LEfSe analysis of fungal (A) and bacterial (B) clades with an LDA threshold of 4.7 for fungi and 3.0 for bacteria; Figure S4: Correlation between the observed variables for fungi and bacteria at the end of litter decomposition under different endophytic fungal colonization treatments.
