# Supplementary material for: Priority Colonization of Endophytic Fungal Strains Drives Litter Decomposition and Saprotroph Assembly via Functional Trait Selection in Karst Oak Forests
Source: Microorganisms. 2025 May 3;13(5):1066. doi: 10.3390/microorganisms13051066 (PMC12114589; doi:10.3390/microorganisms13051066)
Supplement: Supplementary file 1 [file microorganisms-13-01066-s001.zip › microorganisms-3580992-supplementary.pdf]

Priority Effects of Endophytic Fungi Drive Decomposition and Saprotroph Assembly in Karst Oak Leaf Litters

Dongmei Yang[1,2], Zaihua He[1,2], Yonghui Lin[1,2,*], Xingbing He[1,2,*] and Xiangshi Kong[3]

[1]College of Biology and Environmental Sciences, Jishou University, Jishou 416000, China

[2]Hunan Provincial key Laboratory of Ecological Conservation and Sustainable Utilization of Wulingshan Resources, Jishou University, Jishou 416000, China

[3]College of Tourism and Management Engineering, Jishou University, Zhangjiajie 427000, China

[*]Correspondences:

Yonghui Lin, linyonghui@jsu.edu.cn; Xingbing He, hexb@jsu.edu.cn

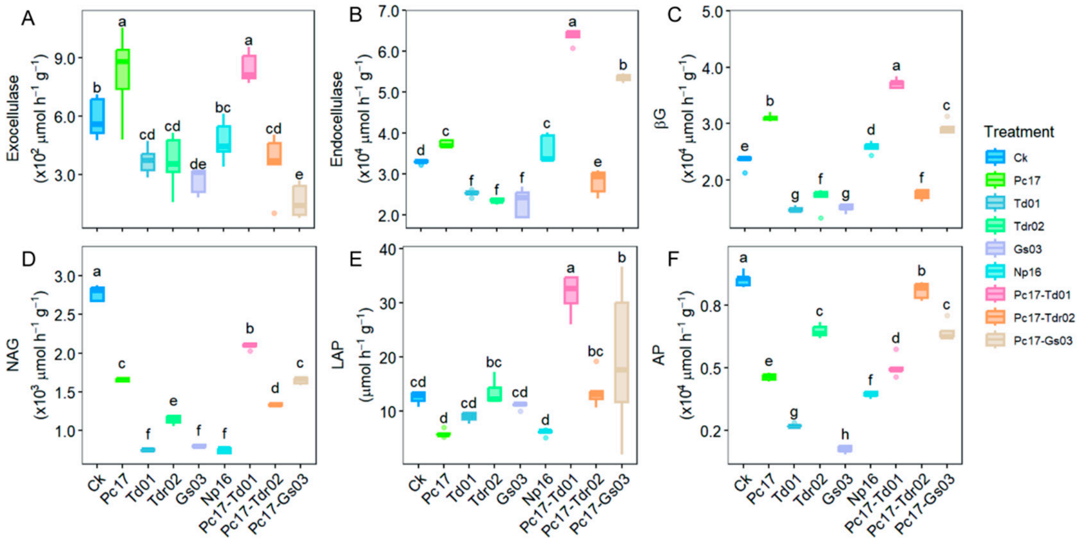

Figure S1. Changes in the activity of single extracellular enzymes at the end of litter decomposition under different endophytic fungal colonization treatments. βG, β-1,4-glucosidase; NAG, β-1,4-N-acetylglucosaminidase; LAP, leucine aminopeptidase; AP, acid phosphatase. Different lowercase letters indicate significant differences ($p < 0.05$, Duncan's test) among treatments.

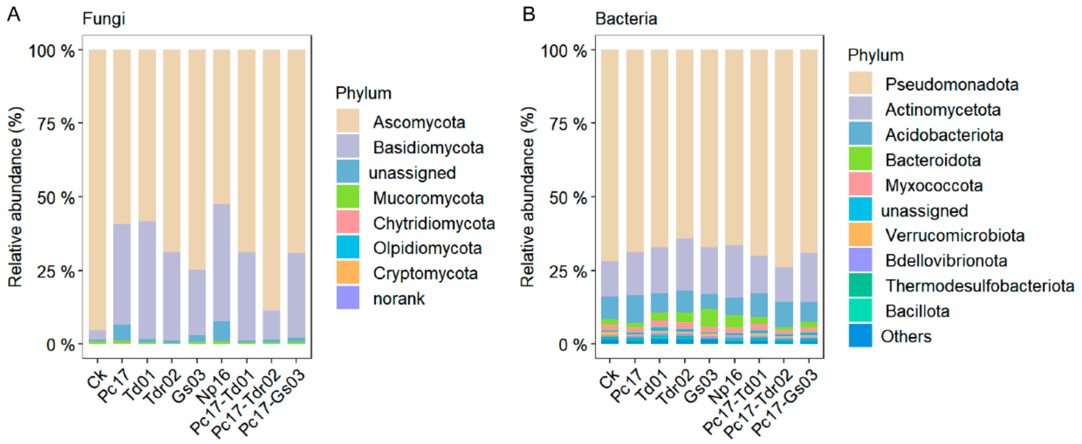

Figure S2. Species composition of the top 10 taxa at the phylum level of fungal(A) and bacterial communities(B) under different endophytic fungal colonization treatments.

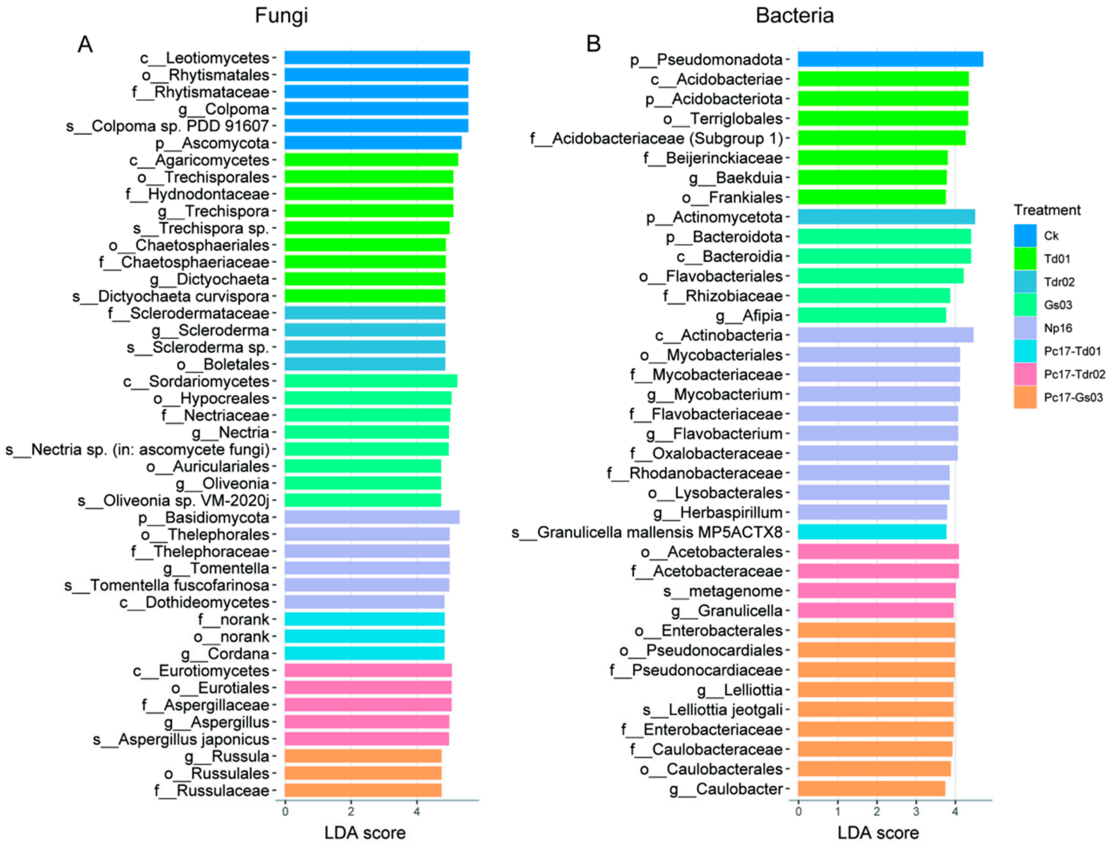

Figure S3. LEfSe analysis of fungal (A) and bacterial (B) clades with an LDA threshold of 4.7 for fungi and 3.0 for bacteria.

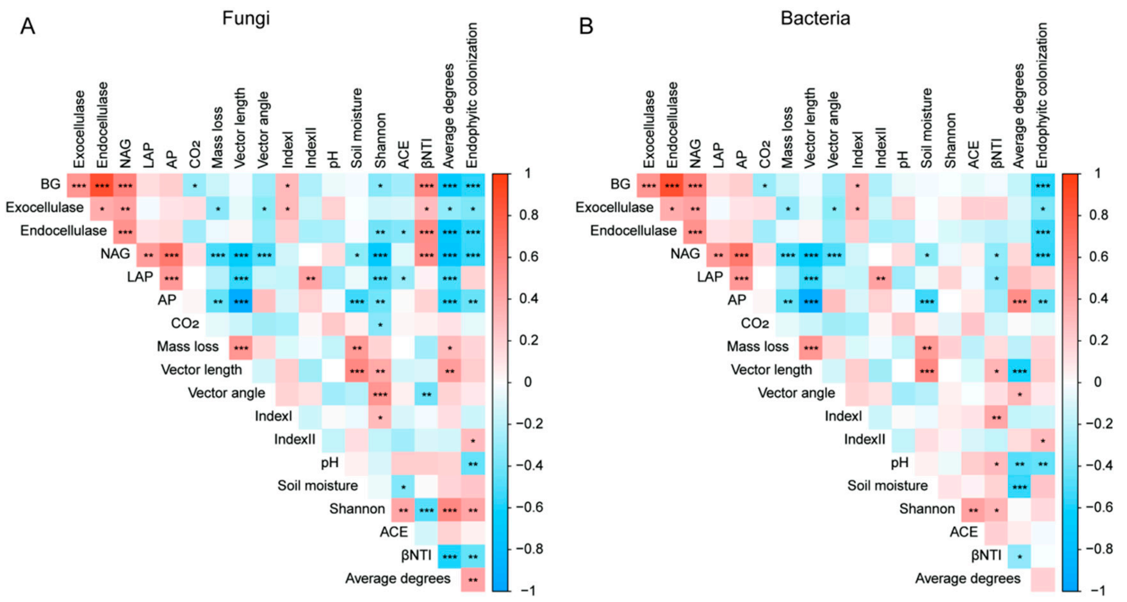

Figure S4. Correlation between the observed variables for fungi and bacteria at the end of litter decomposition under different endophytic fungal colonization treatments. βG, β-1,4-glucosidase; NAG, β-1,4-N-acetylglucosaminidase; LAP, leucine aminopeptidase; AP, acid phosphatase. *$p < 0.05$, **$p < 0.01$, ***$p < 0.001$ indicate significant differences.
